# Some Guidelines for the Synthesis and Melting Characterization of Azide Poly(ethylene glycol) Derivatives

**DOI:** 10.3390/polym12061269

**Published:** 2020-06-02

**Authors:** Daniel González-Fernández, Mercedes Torneiro, Massimo Lazzari

**Affiliations:** 1 Departamento de Química Física, Facultade de Química, and Centro Singular de Investigación en Química Biolóxica e Materiais Moleculares (CIQUS), Universidade de Santiago de Compostela, 15782 Santiago de Compostela, Spain; d_a_g_d_a@hotmail.com; 2 Departamento de Química Orgánica, Facultade de Química, Universidade de Santiago de Compostela, 15782 Santiago de Compostela, Spain

**Keywords:** poly(ethylene glycol), PEGylation, nanoparticles, functionalization, azide-terminated, melting behavior

## Abstract

We provide fundamental guidelines in the form of a tutorial to be taken into account for the preparation and characterization of a specific class of poly(ethylene glycol) (PEG) derivatives, namely azide-terminated PEGs. Special attention is given to the effect of these chain end groups and their precursors on properties affecting the PEGylation of proteins, nanoparticles and nanostructured surfaces. Notwithstanding the presence of ^13^C satellite peaks, we show that ^1^H NMR enables not only the routine quantitative determination of chain-end substitution, but is also a unique method to calculate the absolute number average molecular weight of PEG derivatives. In the use of size exclusion chromatography to get molecular weight distributions, we highlight the importance of distinguishing between eventual secondary reactions involving molecular weight changes and the formation of PEG complexes due to residual amounts of metal cations from reactants. Finally, we show that azide end groups affect PEG melting behavior. In contrast to oxygen-containing end groups, azides do not interact with PEG segments, thus inducing defect formation in the crystal lattice and the reduction of crystal sizes. Melting temperature and degree of crystallinity decrease become especially relevant for PEGs with very low molecular weight, and its comprehension is particularly important for solid-state applications.

## 1. Introduction

Poly(ethylene glycol) (PEG) is a semicrystalline polymer with tremendous applications in biological contexts and for industrial uses [1,2]. Its properties of water-solubility, non-toxicity, biocompatibility and nonimmunogenicity are very advantageous for biomedical and pharmaceutical purposes [3,4]. The incorporation of PEG-containing molecules (so-called PEGylation) into peptides, proteins and drug delivery systems improves their bioavailability and solubility under physiological conditions, enhances resistance to protein degradation and reduces antigenicity [5,6,7]. The preparation of PEG-modified molecules, nanoparticles or nanostructured surfaces is commonly performed via reactive groups, such as aldehyde, amine, thiol or azide, complementary to some substrate target functions [8,9,10,11]. In the case of using PEG as a modifier, good control of the efficiency of functionalization is therefore fundamental, as well as the knowledge of the effect of the introduction of new end groups on fundamental properties and how such groups may affect characterization methodologies. For other solid-state applications such as in polymer electrolytes, ionic liquids or supercapacitors [12,13,14] or in hydrogel scaffolds for tissue engineering [15], other PEG characteristics—especially crystalline morphology and molecular weight—become important, including how they are influenced by molecular and structural changes [16,17,18].

In view of the lack of consistency in the analytical approach and data interpretation that is sometimes found in publications in this field, in the present work we intended to provide fundamental guidelines for the preparation and characterization of a specific class of PEG derivatives suitable for PEGylation, namely, mono- and bifunctional azide-terminated PEGs, with special attention to the effect of these chain end groups and their precursors on properties affecting their application. After proposing an optimization of the synthetic procedure, in the first part we offer practical information in the form of a tutorial, with practical information on difficulties that may arise during nuclear magnetic resonance (NMR), matrix-assisted laser desorption/ionization time-of-flight mass spectrometry (MALDI-TOF MS) and size exclusion chromatography (SEC) analysis. For each characterization technique, we show examples of critical points, with special attention on the (mis)interpretation of results. In the second part, we introduce and discuss an often-neglected aspect related to the morphological characterization of PEGs—namely, the effect of molecular weight and the unexpected influence of different functional groups as chain ends on the melting behavior. These characteristics are particularly important for the use of PEG as a coupling agent for soft-matter applications.

## 2. Materials and Methods

### 2.1. Materials

Mesyl chloride (MsCl, Merck, Darmstadt, Germany, 99.8%), sodium azide (NaN_3_, Sigma Aldrich, Merck, Darmstadt, Germany, 99.5%,), ethanol (AP Medical, Barcelona, Spain, 99%) and tetrahydrofuran (THF, Fischer Sci., Pittsburgh, PA, USA, 99.8%) were used as received. Methylene chloride (CH_2_Cl_2_, Fischer Sci., Pittsburgh, PA, USA, 99.9%) and triethylamine (Et_3_N, Merck, Darmstadt, Germany, 99%) were distilled prior to use from calcium hydride and phosphorus pentoxide, respectively. α-Methoxy-ω-hydroxy PEG with nominal molecular weight 350 Da (mPEG_350_-OH), α,ω-dihydroxy PEG with nominal molecular weight 400 Da (HO-PEG_400_-OH), mPEG_550_-OH and mPEG_2000_-OH were purchased from Merck, Darmstadt, Germany) while HO-PEG_2100_-OH, mPEG_2400_-OH, mPEG_5600_-OH, HO-PEG_7800_-OH, mPEG_11000_-OH and HO-PEG_13000_-OH were purchased from Polymer Source (Montreal, QC, Canada). All the polymers were used as received.

### 2.2. Synthesis

#### 2.2.1. Typical Procedure for the Synthesis of Mesylate PEG

Dry mPEG_350_-OH (0.511 g, 1.46 mmol, 1 eq.) was placed in an oven-dried flask under dry argon (L-50) flow and dissolved in CH_2_Cl_2_ (15 mL) before the addition of Et_3_N (260 mL, 1.9 mmol, 1.33 eq.). The mixture was cooled in an ice-salt bath at −10 °C prior to the addition of MsCl (25 mL, 3.2 mmol, 2.1 eq.), and allowed to warm at room temperature under stirring over 12 h. The reaction mixture was then diluted with water (10 mL) and extracted with CH_2_Cl_2_ (3 × 20 mL). The organic phase was washed with brine (3 × 20 mL), while the aqueous phase was extracted with CH_2_Cl_2_ (3 × 20 mL). The combined organic solution was finally dried over anhydrous Na_2_SO_4_, filtered, and concentrated using a rotary evaporator to obtain a viscous liquid (mPEG_350_-OMs, 0.611 g, 1.42 mmol, 99% yield). ^1^H NMR (CDCl_3_, 500 MHz): δ 4.35 (2H, m, MsOC**H_2_**), 3.73 (2H, m, MsOCH_2_C**H_2_**), 3.61 ((4xn + 2)H, s, (C**H_2_**C**H_2_**O)n + C**H_2_**CH_2_OCH_3_), 3.52 (2H, m, C**H_2_**OCH_3_), 3.35 (3H, s, OCH_3_), 3.03 (3H, s, MsO).

An identical procedure was used for dimesylate PEGs, increasing the amount of MsCl to 4 eq. Mesylate PEGs with molecular weight higher than 2000 Da were obtained as solid residues.

#### 2.2.2. Typical Procedure for the Synthesis of Azide PEG

Dry mPEG_350_-OMs (0.611 g, 1.42 mmol) was placed in a flask under dry argon (L-50) flow and dissolved in ethanol (30 mL) before the addition of NaN_3_ (0.139 g, 2.141 mmol, 1.5 eq.). The mixture was submitted to reflux for 12 h. After cooling to room temperature, the solution was concentrated on a rotary evaporator and dissolved in CH_2_Cl_2_ (20 mL). The organic solution was finally dried over anhydrous Na_2_SO_4_, filtered and concentrated using a rotary evaporator to obtain a viscous liquid (mPEG_350_-N_3_, 0.518 g, 1.38 mmol, 97% yield). ^1^H NMR (CDCl_3_, 500 MHz): δ 3.68–3.64 ((2 + 4xn + 2)H, m, N_3_CH_2_C**H_2_** + (C**H_2_**C**H_2_**O)n + C**H_2_**CH_2_OCH_3_), 3.54 (2H, m, C**H_2_**OCH_3_), 3.40–3.37 ((2 + 3)H, m, N_3_CH_2_ + OCH_3_).

An identical procedure was used for diazide PEGs, increasing the amount of NaN_3_ to 2.5 eq. Azide PEGs with molecular weight higher than 2000 Da were obtained as solid residues.

### 2.3. Characterization

NMR spectra were recorded in CDCl3 on a Bruker DRX (Bruker, Billerica, MA, USA) at 500 MHz. Chemical shifts were reported on the δ scale (ppm) downfield from tetramethylsilane (δ = 0.0 ppm) using the residual solvent signal at δ = 7.26 ppm (1H, CDCl3) as the internal standard. MALDI-TOF mass spectra were recorded on a Bruker Ultraflex III TOF/TOF spectrometer (Bruker, Billerica, MA, USA) using CDCl_3_ as the solvent and dithranol/AgTFA as the matrix.

SEC analyses were performed by using a PL-GPC 50 (Agilent, Santa Clara, CA, USA) apparatus equipped with a MIXED-E column (3 µm, 7.5 mm × 300 mm) with a nominal exclusion limit of 30,000 Da and a high resolution for low molecular weights. Sample solutions of approximately 0.1% (*w*/*v*) concentration were prepared in THF (stabilized with 200 ppm 2,6-dibutyl-4-methylphenol), which was also used as an eluent at a flow rate of 1 mL·min^−1^, at 40 °C. A refractive index detector was used, and column calibration was performed with PEG narrow distribution standards. A third-order polynomial equation was obtained from the regression analysis.

DSC thermograms were obtained with a Q200 (TA Instruments, New Castle, DE, USA) calorimeter equipped with a refrigerated cooling system in the temperature range from 0 to 90 °C, using 3–5 mg samples with a scanning rate of 20 °C·min^−1^, under a 50 mL·min^−1^ nitrogen flow.

## 3. Results and Discussion

**Scheme 1:**
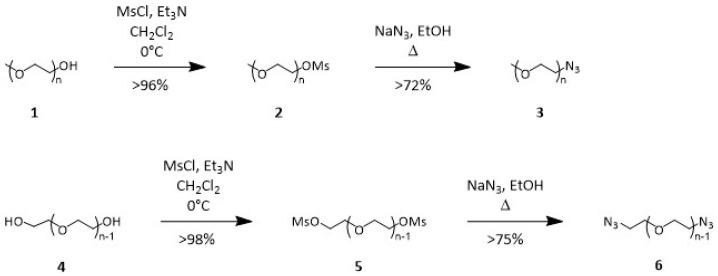
Synthesis of mono- and bifunctional azide-terminated PEG.

A series of α-methoxy-ω-hydroxy and α,ω-dihydroxy PEGs with nominal molecular weight in the range of 350–13,000 Da and very narrow molecular weight distribution, polydispersity index (PDI) < 1.05, were transformed in their corresponding azide-terminated PEGs. Mono- and bifunctional azide PEG derivatives could then be used, as they are in the field of bioconjugate chemistry for PEGylation of natural or synthetic molecules and macrostructures (proteins, antibodies, dendrimers etc.) [8,9,19] or transformed in other reactive groups, such as for the preparation of amines as linking agents for reductive amination reactions suitable for chemical, surface or particle modifications [5,20,21]. The two-step reaction shown in Scheme 1 consists of the transformation of the hydroxyl terminals in **1** or **4** into the corresponding azide **3** or **6** through the mesylate intermediates, refining previously reported conditions [9,19]. The mesylate intermediates **2** and **4** were prepared in CH_2_Cl_2_ with Et_3_N and mesyl chloride excess at −10 °C. Mesylates were displaced with excess NaN_3_ and 1.5 or 2.5 equivalents of mono- or bifunctional intermediates, respectively, in refluxing ethanol. Yields are summarized in Table 1.

**Figure 1:**
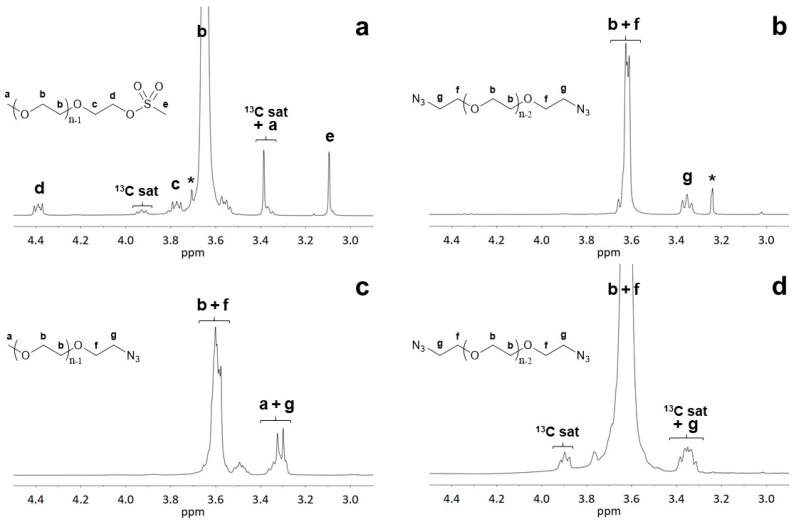
^1^H NMR spectra of mPEG_2000_-Oms (**a**) and N_3_-PEG_400_-N_3_ (**b**), mPEG_350_-N_3_ (**c**), N_3_-PEG_7800_-N_3_ (**d**). Mark (*) denotes signal due to residual methanesulfonic acid or undeuterated solvents.

The quantitative functionalization of purified products can be confirmed by ^1^H NMR (substitution > 99% for all the polymers). At the same time, it is possible to take advantage of an often underutilized potential of NMR when applied to polymers with controlled molecular structure and chain ends, namely, the determination of the number of repeating units [22,23,24], and therefore of the number average molecular weight, *M_n_* (Table 1). In principle, the introduction of new groups may be followed by the appearance of specific signals, such as those of methyl protons of mesyl groups (Figure S1 in Supplementary Materials) or from the –C*H*_2_–N_3_ methylene protons. Unfortunately, possible overlapping complicates peak integration. In particular, the carbon satellites of the main methylene peak at 4.0–3.9 ppm and 3.4–3.3 ppm, partially overlapped with the C*H*_3_ signal of methoxy PEG at 3.37 ppm or with the –C*H*_2_–N_3_ methylene proton signal centered at 3.35 ppm (Figure 1). Such satellite peaks resulted from the coupling of ^1^H atoms to an adjacent ^13^C atom and had an intensity that was 0.54% of the parent peak, therefore being especially challenging for the identification and quantification of minor signals [25]. Suppression of ^13^C satellite peaks is possible through different decoupling experiments, usually at a high cost of selectivity or sample heating, and is not usually worth the effort for routine measurements. In the case of functionalized chain end PEGs, the limitation is imposed by the relative satellite intensity with respect to the intensity of the target functional group signals, which practically hinders precise calculations for polymers with a molecular weight higher than 1000 Da.

The impossibility of integrating the signal due to methoxy chain ends does not prevent calculation of the length of PEG macromolecules, since the number of repeating units may be obtained from the integral intensity of the proton signals of the methylene-oxy –CH_2_–C*H*_2_–O to that of methylene protons adjacent to the other chain end group. Conversely, a different strategy may be applied for azide PEG derivatives, exploiting their reduction to amino derivatives, with distinctly different NMR signals (e.g., Figure S2). The broad –N*H*_2_ signal centered at 2.75 was far away from any other interfering signal in both mono- and bifunctional amino PEGs and allowed for the estimation of chain lengths. For all the polymers, the values were coincident with those obtained from mesylated molecules.

**Figure 2:**
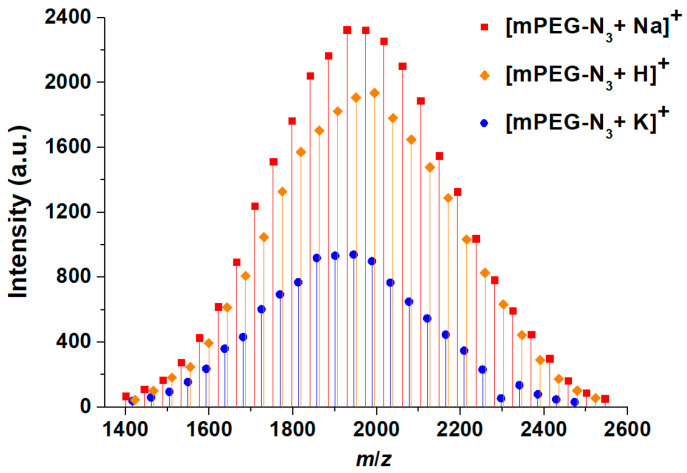
MALDI-TOF mass spectrum of mPEG_2000_-N_3_ adduct distributions.

MALDI-TOF MS is an exact tool for the determination of absolute molecular weights, and offers an accurate evaluation of effective functionalization through the detection of specific ions (example in Table 2 and Figure 2 for mPEG_2000_-N_3_). In all the samples, three apparent distributions could be seen, but closer examination suggested that there was only one polymer distribution, with three different cationization routes. In all the series, the peaks were separated by 44 Da, corresponding to the repeating unit mass. The series corresponded to the protonated forms as well as Na and K adducts of the PEG oligomers. In our case, the values of polymers with the lowest molecular weights were determined and were in good agreement with those from NMR (Table 1). On the other hand, as sometimes occurs with this technique, results from samples with higher molecular weights were matrix dependent. In our opinion, the fine-tuning of analysis conditions for any single type of PEG is too time consuming and would not be compatible with the guidelines we are proposing for a routine characterization to follow functionalization efficiency.

**Figure 3:**
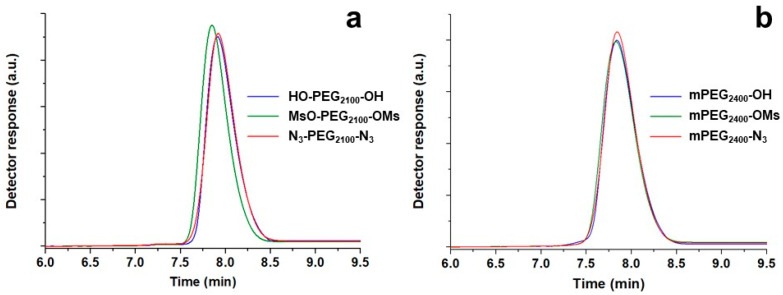
Normalized SEC curves of mPEG_2000_ (**a**) and PEG_2400_ (**b**) series.

SEC is an excellent method of disclosing eventual unexpected reactions such as coupling or any other process involving molecular changes. In its absence, almost overlapping chromatograms of polymers before and after functionalization must be observed, without the appearance of any shoulder or secondary peaks, as exemplified in Figure 3. Interestingly, the method was also able to detect minimal differences in the solution behavior, especially when PEGs underwent a double substitution (Table 3).

In SEC, separation occurs because of the hydrodynamic molecular volume, and average molecular weights and their distributions are calculated through a calibration with opportune standard samples with well-known molecular weights and very narrow distributions. A small modification of terminals affecting the molecular sizes may therefore induce apparent molecular weight changes, particularly for short chains. Hydroxy and azide derivatives had a molecular weight very similar to each other, and the apparent values deduced from SEC measurements were almost identical. The mesylated intermediates, in which the replacement of a hydroxyl hydrogen for a mesyl group entailed a formal 78 Da increase, showed a higher than expected apparent value (e.g., a 350 Da increase for Ms-PEG_2100_-Ms). This result did not imply secondary structural changes, but was due to conformational effects in the solution that arose from the steric bulkiness of such substituent groups, whose effect became particularly relevant for the smallest molecules [26]. This hypothesis was also confirmed by the fact that SEC systematically overestimated the *M_n_* of PEGs, possibly due to the common use of linear dimethoxy PEGs as calibration standards. The presence of such terminal groups, which underwent less interaction with the chromatographic solvent than the hydroxyls, for example, entailed more compact conformations in solution. In the case of the analysis of functionalized PEGs, their greater than predictable hydrodynamic volumes with respect to standard “neutral” dimethoxy PEGs ultimately corresponded to shorter elution times, and therefore to larger apparent molecular weights.

**Figure 4:**
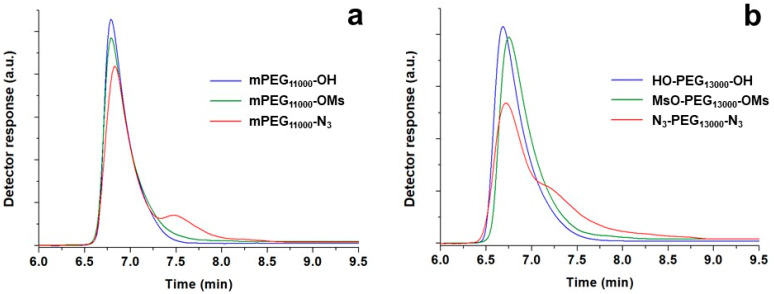
Normalized SEC curves of mPEG_11000_ (**a**) and PEG_13000_ (**b**) series.

At the end, it is essential to mention the appearance of shoulders or even a secondary peak at longer retention times in the SEC curves as a consequence of mesylate displacement, especially for PEGs with molecular weight >5000 Da. The secondary peaks, such as those visible in the chromatograms in Figure 4, became progressively more relevant with the increase of the molecular weight and were associated to the complexation properties of PEG chains [27]. PEG in solution easily forms pseudo-crown ether complexes (podands) with different cations. The presence of trace amounts of residual sodium cations from NaN_3_ used during azide functionalization may justify the formation of some agglomerates with formation constant proportional to chain length [27] and more compact conformations than under pristine conditions.

It is well known that PEG is a highly crystalline polymer with a melting temperature (*T_m_*) of 65–67 °C and a degree of crystallinity (*X*_c_) up to 95%, at least when the molecular weight is high enough to ignore molecular and structural influences. A typical DSC thermogram is shown in Figure S3. After the first heating cycle, which is usually affected by some physical aging, all the second and subsequent heating and cooling profiles showed reproducible behavior, and the corresponding values were considered as relevant.

**Figure 5:**
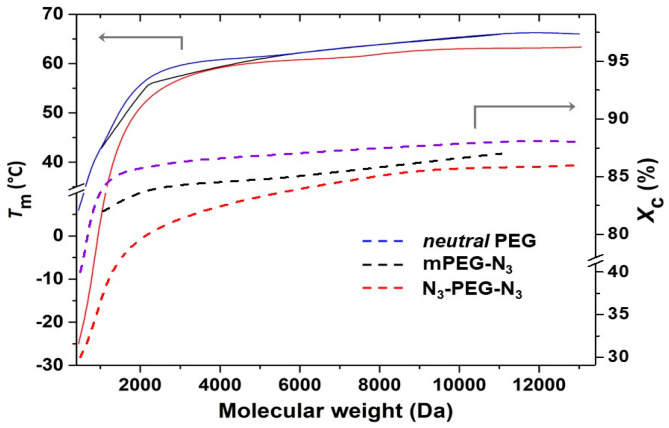
Dependence of *T_m_* and *X_c_* on the molecular weight of neutral PEG, mPEG-N_3_ and N_3_-PEG-N_3_ series.

As observed in many polymers and in lower-molecular-weight PEGs, both general morphological properties and more specifically, *X*_c_, depend on molecule length [28]. Concerning chain ends, PEGs such as those usually obtained from anionic polymerization (i.e., α,ω-dihydroxy PEGs (in this context named as neutral)) and those with one or two methoxy chain ends show properties very similar to each other, with negligible effects due to different combinations of different hydroxyl and methoxy end groups [29]. Some effect on crystallinity is expected only in the presence of more voluminous or chemically different chain ends. Figure 5 shows *T_m_* and *X*_c_ of mono- and bifunctional azide-terminated PEGs as a function of the molecular weight, compared with the values of neutral PEGs. Values were calculated using a Δ$H_{m}^{0}$ = 196.8 J g^−1^ [30]. As for non-functionalized PEGs, both azide PEG derivative series showed an increase of *T_m_* and *X*_c_ for increasing molecular weights, although with substantial differences. In mono azide derivatives, the substitution of the OH group by an azide seemed to have a very limited effect on crystallinity. On the other side, the presence of two azide groups reduced both *T_m_* and *X*_c_. At first glance, the shorter the PEG macromolecules, the bigger the perturbation of the crystals. If for N_3_-PEG_13000_-N_3_, *T_m_* and *X*_c_ were a little lower than expected for such molecular weight, in N_3_-PEG_2100_-N_3_, *X*_c_ = 80%, in comparison with 85% for the corresponding HO-PEG_2100_-OH. In the case of the smallest N_3_-PEG_400_-N_3_, melting occurred at a temperature more than 20 °C lower than for HO-PEG_400_-OH, with *X*_c_ decreasing from 40% to 30%. A similar behavior was observed for the temperature of crystallization and the corresponding *X*_c_.

The effect of molecular weight on glass transition has been studied for a long time, and the dependence of the glass transition temperature upon the chain length is expressed by the Flory–Fox equation, of general applicability for various types of polymers [31]. On the contrary, the extent of the effect of polymer chain length on the melting behavior depends upon whether the chain ends participate in the crystal formation. In PEGs with molecular weight up to approximately 4000 Da, extended chain crystals with monoclinic structures are formed [32], without the participation of chain ends. However, the fact that unit-cell structure and crystalline parameters are slightly affected by the molecular weight [33] suggests an influence of end groups in the formation of defects in the crystal lattice [29]. Shorter crystallizable segments would induce the formation of smaller crystals and the consequent depression in the melting point, whereas the size and the level of perturbation of the terminal groups reduce the *X*_c_ even more. Methoxy, hydroxy and other O-containing end groups are not expected to perturb the crystal lattice, apart from limiting the length of crystallizable chain segments. In contrast, groups such as the azides, not interacting with adjacent segments (e.g., through the formation of hydrogen bonds) affect further crystallization and melting behaviors. Alternatively, Cheng et al. [29] proposed that bulkier end groups hamper the transformation kinetic to a more stable crystalline structure from initially formed and less-perfect crystals which show lower *T_m_* and at the same time result in smaller overall crystallinity.

A similar effect on PEG crystallization has already been discussed for some graft and block copolymers, and more in general for PEG conjugates [34], where the steric constraints imposed by the linked chains affected the amount and the structure of crystals and should be taken into account in all cases where PEG chains are then attached to other macromolecules.

## 4. Conclusions

The guidelines herewith discussed should be considered as practical suggestions to be taken into account for the synthesis and careful characterization of azide PEG derivatives, and may also be used as a starting point for the precise detection of molecular and structural properties of PEGylated substrates. After proposing an optimization of the synthesis procedure, we showed that ^1^H NMR enabled not only the customary follow up of PEG modification and quantitative determination of chain-end substitution as expected, but was also an efficient method to calculate the absolute number-average molecular weight of PEG derivatives. Although in principle MALDI-TOF MS is also a precise tool for the determination of absolute molecular weights, the matrix dependence of results and the need for time-consuming fine-tuning of analysis conditions for each series of PEGs, possibly due to the presence of traces of functionalization reactants, makes this technique incompatible with routine characterization.

SEC analysis can easily disclose eventual secondary reactions taking place during PEG functionalization, such as coupling or any other process involving molecular weight changes. We also drew attention to the fact that the hypothetical formation of reaction products with apparent molecular weights lower than those of the initial PEG was sometimes an indicator of the presence of residual amounts of metal cations from reactants, which induced the formation of complexes with podands in PEG. Such complexes had smaller hydrodynamic volumes than free PEG chains and therefore eluted at longer retention times. Finally, particular attention should also be paid to melting characterization, as we showed that end groups might affect *T_m_* and *X*_c_. Differently from methoxy, hydroxy and other O-containing end groups, azides do not interact with PEG segments, thus inducing defect formation in the crystal lattice and the reduction of crystal sizes. *T_m_* and *X*_c_ decreases become especially relevant for PEGs with very low molecular weight, and are particularly crucial for solid-state applications.

## Figures and Tables

**Table 1:** Summary of functionalization efficiency and molecular characteristics obtained from ^1^H NMR.

Polymer	-Ms Yield ^a^ (%)	-N_3_ Yield ^a^ (%)	Theoretical Repeating Units ^b^	PEG-OMs Repeating Units ^c^	PEG-N_3_ Repeating Units ^d^
mPEG_350_-OH	99	97	7	7	7
HO-PEG_400_-OH	99	99	9	9	9
mPEG_550_-OH	99	90	12	12	12
mPEG_2000_-OH	99	96	45	45	-
HO-PEG_2100_-OH	99	88	47	47	-
mPEG_2400_-OH	99	76	54	54	-
mPEG_5600_-OH	99	72	127	140	-
HO-PEG_7800_-OH	98	75	177	200	-
mPEG_11000_-OH	99	89	249	170	-
HO-PEG_13000_-OH	99	91	295	311	-

^a^ Yield refers to the isolated and purified product. ^b^ Whole number of repeating units from molecular weight provided by the manufacturer. ^c^ Whole number of repeating units of mesylated derivatives as calculated by ^1^H NMR. ^d^ Whole number of repeating units of azide derivatives as calculated by ^1^H NMR.

**Table 2:** Summary of functionalization efficiency and molecular characteristics obtained from ^1^H NMR.

Adduct Series	*M_n_* ^a^	*M_n,MALDI_* ^b^	PDI ^b^
[mPEG_2000_-N_3_ + H]^+^	2040	2020	1.01
[mPEG_2000_-N3 + Na]^+^	2060	2010	1.01
[mPEG_2000_-N_3_ + K]^+^	2080	1980	1.01

^a^ Theoretical value calculated from molecular weight provided by the manufacturer. ^b^ Calculated by MALDI-TOF.

**Table 3:** Molecular characteristics for some functionalized PEG families as obtained by SEC.

Polymer	*M_n_*	*M_n,SEC_* ^b^	PDI ^b^
HO-PEG_2100_-OH	2100	2530	1.03
MsO-PEG_2100_-OMs	2260 ^a^	2880	1.03
N_3_-PEG_2100_-N_3_	2150 ^a^	2540	1.03
mPEG_2400_-OH	2400	2780	1.04
mPEG_2400_-OMs	2480 ^a^	2880	1.03
mPEG_2400_-N_3_	2430 ^a^	2800	1.03
mPEG_5600_-OH	5600	6320	1.05
mPEG_5600_-OMs	5680 ^a^	6480	1.05
mPEG_5600_-N_3_	5630 ^a^	6170	1.04
HO-PEG_7800_-OH	7800	8380	1.06
MsO-PEG_7800_-OMs	7960 ^a^	8260	1.08
N_3_-PEG_7800_-N_3_	7850 ^a^	7800	1.08

^a^ Theoretical value calculated from molecular weight provided by the manufacturer. ^b^ Calculated by SEC.

## Supplementary Materials

The following are available online at https://www.mdpi.com/2073-4360/12/6/1269/s1, Figure S1: 1H NMR spectra of MsO-PEG400-OMs and mPEG550-OMs, Figure S2: 1H NMR spectrum of NH2-PEG400-NH2, Figure S3: DSC curves of mPEG_2400_-N_3_.
