# Supplementary material for: Some Guidelines for the Synthesis and Melting Characterization of Azide Poly(ethylene glycol) Derivatives
Source: Polymers (Basel). 2020 Jun 2;12(6):1269. doi: 10.3390/polym12061269 (PMC7362184; doi:10.3390/polym12061269)
Supplement: Supplementary file 1 [file polymers-12-01269-s001.pdf]

polymers

Supplementary Materials

Some Guidelines for the Synthesis and Melting Characterization of Azide PEG Derivatives

Daniel González-Fernández [1,2], Mercedes Torneiro [2,*] and Massimo Lazzari [1,*]

[1] Departamento de Química Física, Facultade de Química, and Centro Singular de Investigación en Química Biolóxica e Materiais Moleculares (CIQUS), Universidade de Santiago de Compostela, 15782 Santiago de Compostela, Spain; d_a_g_d_a@hotmail.com

[2] Departamento de Química Orgánica, Facultade de Química, Universidade de Santiago de Compostela, 15782 Santiago de Compostela, Spain

* Correspondence: massimo.lazzari@usc.es (M.L.); mercedes.torneiro@usc.es (M.T.)

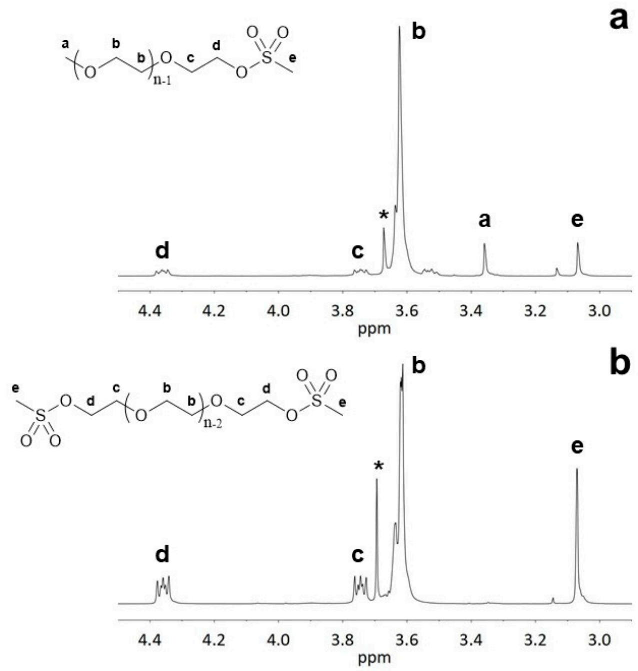

Figure S1. ^{1}H NMR spectra of MsO-PEG400-OMs (**a**) and mPEG550-OMs (**b**). Mark denotes signal due to residual methanesulfonic acid.

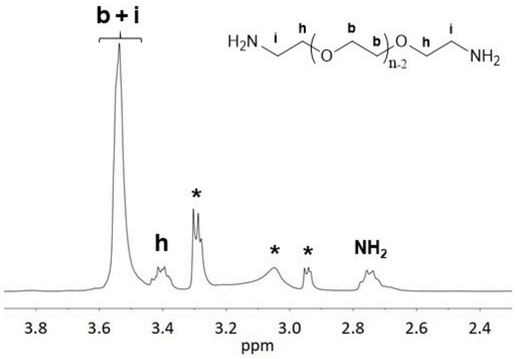

Figure S2. ^{1}H NMR spectrum of NH_2-PEG400-NH_2. Marks denote signals due to residual undeuterated solvents.

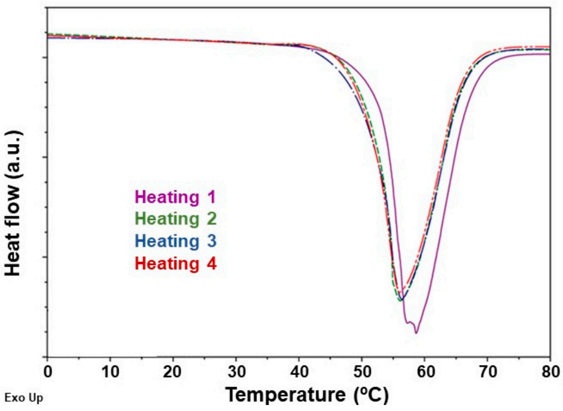

Figure S3. DSC curves of mPEG2400-N_3, heating cycles 1-4.
